# Dataset on the global distribution of shallow groundwater

**DOI:** 10.1016/j.dib.2023.108973

**Published:** 2023-02-13

**Authors:** Mehmet Evren Soylu, Rafael L. Bras

**Affiliations:** School of Civil and Environmental Engineering, Georgia Institute of Technology, Atlanta, GA, USA

**Keywords:** Shallow groundwater, Remote sensing, Soil moisture, SMAP

## Abstract

Shallow groundwater (GW), defined as the water table of unconfined or perched aquifers that is near enough to the land surface to influence the vadose zone and the surface soil moisture, impacts land surface water, energy, and carbon cycles by providing additional moisture to the root zone via capillary fluxes. Although the interactions of shallow GW and the terrestrial land surface are widely recognized, incorporating shallow GW into the land surface, climate, and agroecosystem models is not yet possible due to the lack of groundwater data. Groundwater systems are affected by various factors, including climate, land use/land cover, ecosystems, GW extractions, and lithology. Although GW wells are the most direct and accurate way of monitoring water table depths at point scales, upscaling GW levels from point scale to areal or regional scale poses significant challenges.

Here, we provide high spatiotemporal resolution global maps of the terrestrial land surface areas influenced by shallow GW from mid-2015 to 2021 (a separate NetCDF file for each year) in a 9 km spatial and daily temporal resolution. We derived this data from NASA's Soil Moisture Active Passive (SMAP) mission spaceborne soil moisture observations with a temporal resolution of 3 days and approximately 9 km grid resolution. This spatial scale corresponds to SMAP's "Equal Area Scalable Earth" (EASE) grids. The central assumption is that the monthly moving average of soil moisture observations and their coefficient of variation are sensitive to shallow GW regardless of the prevailing climate. We process the Level-2 enhanced passive soil moisture SMAP (SPL2SMP_E) product to detect shallow GW signals. The presence of shallow GW data is calculated by an ensemble machine learning model, which is trained using simulations from a variably saturated soil moisture flow model (Hydrus-1D). The simulations span various climates, soil textures, and lower boundary conditions.

The spatiotemporal distribution of shallow GW data based on SMAP soil moisture observations is provided for the first time with this dataset. The data are of value in a wide variety of applications. The most direct use is in climate and land surface models as lower boundary conditions or as a diagnostic tool to verify model results. Some other applications may include flood risk analyses and regulation, identifying geotechnical issues such as shallow GW-triggered liquefaction, global food security, ecosystem services, watershed management, crop yield, vegetation health, water storage trends, and tracking mosquito-borne diseases by identifying wetlands, among other applications.

**Specifications Table**

**Table:** 

Subject	Hydrology and Water Quality
Specific subject area	Distribution of shallow groundwater presence at a global scale with high resolution between 2015 and 2021
Type of data	NetCDF (network common data form) files
How the data were acquired	NASA's SMAP level 2 enhanced passive soil moisture (SM) observation product is used to estimate shallow groundwater (GW) presence. We applied a supervised ensemble machine learning (EML) model to capture the areas under shallow GW influence. To train the EML model, we used SM simulations mimicking SMAP observations with controlled lower boundary conditions (LBC). The Hydrus-1D model, a variably saturated SM flow model, is used to simulate surface SM. Six different LBC scenarios (from free drainage to 0.5m water table depth) across the globe are simulated. The model is driven by the global potential evapotranspiration dataset generated from the NCEP's GDAS and precipitation from NASA's GPM observations.
Data format	Analyzed
Description of data collection	An ensemble machine learning (EML) method is used for the binary classification of GW presence for the location and temporal period of interest. Daily values of coefficient of variation, minimum, maximum, and average soil moisture in a 30-day moving window are the predictors for the EML model. After the initial EML classification, we applied a data correction scheme to eliminate the frequent GW-state changes due to uncertainties in observations [1].
Data source location [2]	*Institution:* Georgia Institute of Technology *City/Town/Region:* Atlanta, GA *Country:* USA *Latitude and longitude (and GPS coordinates, if possible) for collected samples/data:* Global coverage (latitude and longitude coverage extend from: to -85:85° and -180:180°, respectively); The individual yearly data is available through the following links:
	Shallow GW presence for 2015: https://www.hydroshare.org/resource/9462b23c5e1e46bdae6ef8abcdbed365/data/contents/ShallowGW2015.nc Shallow GW presence for 2016: https://www.hydroshare.org/resource/9462b23c5e1e46bdae6ef8abcdbed365/data/contents/ShallowGW2016.nc Shallow GW presence for 2017: https://www.hydroshare.org/resource/9462b23c5e1e46bdae6ef8abcdbed365/data/contents/ShallowGW2017.nc Shallow GW presence for 2018: https://www.hydroshare.org/resource/9462b23c5e1e46bdae6ef8abcdbed365/data/contents/ShallowGW2018.nc Shallow GW presence for 2019: https://www.hydroshare.org/resource/9462b23c5e1e46bdae6ef8abcdbed365/data/contents/ShallowGW2019.nc Shallow GW presence for 2020: https://www.hydroshare.org/resource/9462b23c5e1e46bdae6ef8abcdbed365/data/contents/ShallowGW2020.nc Shallow GW presence for 2021: https://www.hydroshare.org/resource/9462b23c5e1e46bdae6ef8abcdbed365/data/contents/ShallowGW2021.nc NASA's SMAP Enhanced Level 2 Radiometer Half-Orbit 9 km EASE Grid Soil Moisture: https://nsidc.org/data/spl2smp_e/versions/5
Data accessibility	Repository name: HydroShare Data identification number: 10.4211/hs.9462b23c5e1e46bdae6ef8abcdbed365 Direct URL to data: https://doi.org/10.4211/hs.9462b23c5e1e46bdae6ef8abcdbed365
Related research article	Soylu, M. E. and R. L. Bras, Global Shallow Groundwater Patterns from Soil Moisture Satellite Retrievals, IEEE Journal of Selected Topics in Applied Earth Observations and Remote Sensing, 15, (2022) 89-101, https://doi.org/10.1109/JSTARS.2021.3124892.

## Value of the Data

• An observation-based database representing shallow groundwater presence globally is provided. Given the high spatiotemporal resolution, the shallow GW dataset may contribute to the efforts of integrating GW into surface flux models (e.g., ecosystem, land surface models) as a calibration or diagnostic tool to assess the validity of such models. Moreover, the dataset helps reduce the model biases originating from the additional water added to the root zone due to shallow GW presence.
• The dataset presented here can potentially be helpful in a wide variety of research areas, including flood risk analyses and regulation, identifying geotechnical issues such as liquefaction, global food security, ecosystem services, watershed management, crop yield, vegetation health, water storage trends, and global distributions of wetlands.
• Another, more direct, use of the dataset may be to use it as LBC to the surface flux models. This option may improve the soil moisture flux simulation accuracies while reducing the computational cost of such models. Adopting this shallow GW database based on observations, as a driver to control the model LBC allows modelers to solve the Richards equation in 1-D instead of 3-D without losing the accuracy of results.

## 1 Objective

Shallow GW provides additional water to ecosystems and the land surface. The additional water resulting from the coupling between GW and the surface not only affects evapotranspiration but also has the potential to modify ecosystems and components of water, energy, and carbon cycles. The lack of global-scale shallow GW data with high spatial and temporal resolution hinders our understanding of terrestrial water, energy, carbon, and water cycle components.

The theoretical background on generating the shallow GW dataset was explained in detail in [1]. By sharing this dataset with the public, we hope to contribute to solving some of the shallow GW-related problems shared by the wide variety of researchers from different areas, including hydrology, ecology, hydrogeology, and atmospheric sciences.

## 2 Data Description

Shallow GW data is provided in multidimensional NetCDF file contents. A separate NetCDF file is produced for each year from mid-2015 to 2021. Each data file covers the entire temporal range from January 1st to December 31st, except the file for the year 2015, which covers only between June 1st to December 31st due to SMAP data availability.

The dataset has a “Shallow Groundwater Presence” variable, indicating through a binary variable whether the region is under shallow groundwater influence or not. If this variable equals 1, the given pixels are under shallow GW influence. A pixel value of -9999 value indicates that there is no shallow GW influence or that there is not sufficient soil moisture data available to detect shallow GW presence.

File name: ShallowGW2015.nc

Filetype: NetCDF file

Spatial extent: Latitude: -180:180 Degrees North; Longitude: -85:85 Degrees East

Spatial resolution: 9 km

Temporal coverage: Start date:06/01/2015; End date:12/31/2015

Temporal resolution: 1 day

File name: ShallowGW2016.nc

Filetype: NetCDF file

Spatial extent: Latitude: -180:180 Degrees North; Longitude: -85:85 Degrees East

Spatial resolution: 9 km

Temporal coverage: Start date:01/01/2016; End date:12/31/2016

Temporal resolution: 1 day

File name: ShallowGW2017.nc

Filetype: NetCDF file

Spatial extent: Latitude: -180:180 Degrees North; Longitude: -85:85 Degrees East

Spatial resolution: 9 km

Temporal coverage: Start date:01/01/2017; End date:12/31/2017

Temporal resolution: 1 day

File name: ShallowGW2018.nc

Filetype: NetCDF file

Spatial extent: Latitude: -180:180 Degrees North; Longitude: -85:85 Degrees East

Spatial resolution: 9 km

Temporal coverage: Start date:01/01/2018; End date:12/31/2018

Temporal resolution: 1 day

File name: ShallowGW2019.nc

Filetype: NetCDF file

Spatial extent: Latitude: -180:180 Degrees North; Longitude: -85:85 Degrees East

Spatial resolution: 9 km

Temporal coverage: Start date:01/01/2019; End date:12/31/2019

Temporal resolution: 1 day

File name: ShallowGW2020.nc

Filetype: NetCDF file

Spatial extent: Latitude: -180:180 Degrees North; Longitude: -85:85 Degrees East

Spatial resolution: 9 km

Temporal coverage: Start date:01/01/2020; End date:12/31/2020

Temporal resolution: 1 day

File name: ShallowGW2021.nc

Filetype: NetCDF file

Spatial extent: Latitude: -180:180 Degrees North; Longitude: -85:85 Degrees East

Spatial resolution: 9 km

Temporal coverage: Start date:01/01/2021; End date:12/31/2021

Temporal resolution: 1 day

## 3 Experimental design, materials, and methods

**Fig. 1:**
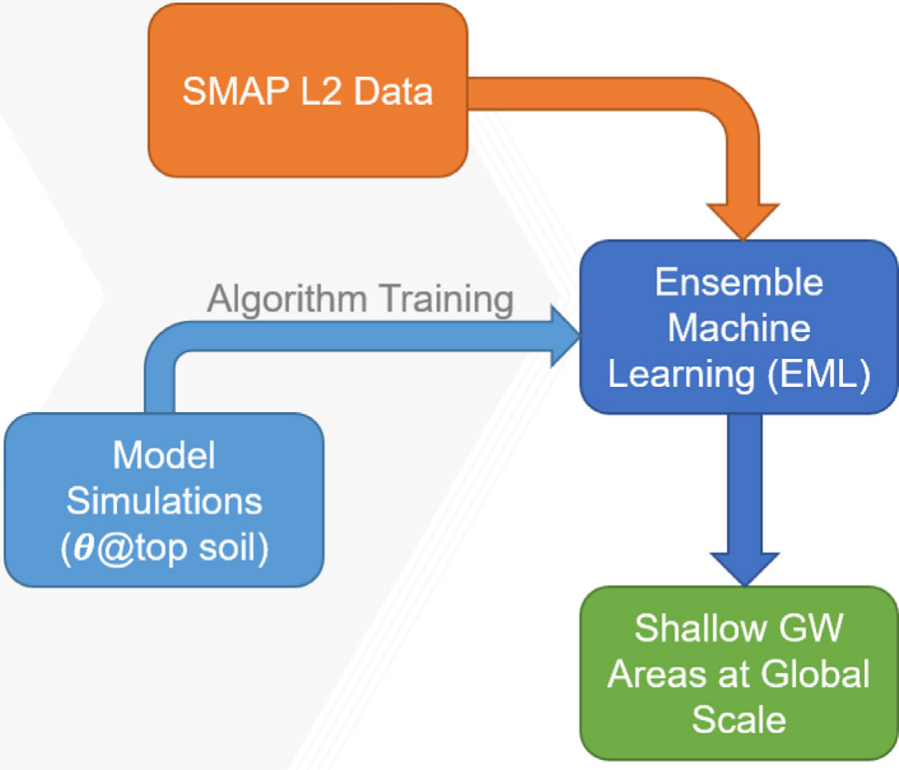
Workflow scheme showing the components to reach the final shallow GW dataset.

To produce the shallow GW dataset, we applied a supervised EML model on SMAP L2 soil moisture observations. Surface soil moisture simulations representing SMAP observations are used to train the EML model (Fig. 1). Below, we provide details about the soil moisture flow model, its inputs, the ensemble machine learning (EML) model, and the SMAP soil moisture observational dataset.

### 3.1 Soil moisture active passive (SMAP) mission observations

The SMAP sensors capture L-band microwave emissions globally with approximately 3 days temporal and 36 km spatial resolution. We use the radiometer-based level 2 enhanced passive soil moisture (SPL2SMP_E Version 5) product, which has a 9 km grid resolution derived from a 36 km spatial resolution [3]. The validity of the SMAP enhanced product has been tested against field observations and compared with other soil moisture retrievals to ensure the consistency and the quality of the SMAP soil moisture products [4], [5], [6]. SMAP satellite makes measurements in the morning (6 A.M. local time) and evening (6 P.M. local time). Generally, the morning overpasses are preferred due to the temperature uniformity across the soil-vegetation column. Nevertheless, evening retrievals have also been shown to be accurate [6]. Both morning and evening SMAP retrievals are used to produce shallow GW dataset.

### 3.2 Soil moisture simulations

The Hydrus-1D model, which solves the Richards equation for variably saturated soil water flow, is used to simulate the surface soil moisture, mimicking SMAP observations. The model is driven by the Global Precipitation Measurement (GPM) mission precipitation data (GPM IMERG Final Precipitation L3 [7]) and the USGS Famine Early Warning System daily global potential evapotranspiration (ETp) dataset generated by the Global Data Assimilation System (GDAS) analysis fields. The ETp dataset is partitioned into potential evaporation and potential transpiration using the MODIS’ enhanced vegetation index (MOD13C2). Potential evaporation is used to calculate the actual water loss from the soil surface, and potential transpiration is used to calculate the plant water uptake within the soil profile by the model via the sink term. We use the van Genuchten soil retention functions [8] in the Hydrus-1D model. The soil hydraulic parameters for the soil retention function are obtained from a global dataset [9].

The Hydrus-1D model is run for 226 points across the globe representing various climate and soil conditions with six different groundwater scenarios applied to each data point. Constant pressure head (Dirichlet) boundary conditions at five different depths, representing constant water table depths from 0.5 m to 2.5 m, in increments of 50 cm, are used. In addition, a simulation with free drainage lower boundary conditions is done. The total soil profile domain thickness is taken as 3 meters, but only the top 5 cm of the simulations are considered to represent SMAP soil moisture observations. The model simulations cover five years period from 2015 to 2019. We use the first year as the model spin-up period. The results of that year are not used in the data analyses.

### 3.3 The machine learning approach

The gentle adaptive boosting (GentleBoost), a variant of the adaptive boosting ensemble machine learning (EML) technique, is used to classify whether the surface soil moisture data is affected by shallow groundwater. To develop the EML method, about 1.2 million soil moisture data points are used in model training, and about 0.8 million data points are used in model testing. The daily coefficient of variation, minimum, maximum, and average surface soil moisture values are used as model predictors. A 30-day moving window average is used to calculate each predictor. We find that using a monthly time window width provides a sufficient number of sample points allowing us to analyze the soil moisture signals in a statistically meaningful way. At the same time, a 30-day window width does not smooth the shallow groundwater signals too much.

Once the EML model is trained, we apply it to the SMAP observations covering the period from mid-2015 to the end of 2021. We adopt a data correction scheme as the last step before the final dataset, as recommended by [10]. The scheme is used to minimize potential errors due to satellite observation uncertainties. The correction scheme calculates the number of consecutive data points representing GW-influenced state and GW-free states. Based on the assumption that the seasonal groundwater influence on soil moisture cannot switch between these two states within short intervals, the sequence of points is reclassified if each GW state is shorter than a certain threshold. A 30-day threshold is selected to properly represent the shallow GW impact on the terrestrial land surface while ensuring the land surface must remain under the influence of one state for a minimum amount of time.

The shallow GW dataset is evaluated against three datasets. First, we compare to baseflow estimations in the southeast US. Second, we check consistency with global scale wetland distributions estimated based on a global scale GW model outputs [11]. Third, we use global scale clay-enriched soil horizon distributions that can lead to perched aquifers as an indicator of potential shallow GW [12]. The comparisons, shown and discussed in detail in [1], indicate that the global distribution of shallow GW dataset introduced here properly detects large riparian corridors, wetlands, and seasonally inundated lowlands.

## Ethics Statements

The current work does not involve human subjects, animal experiments, or any data collected from social media platforms and meets the ethical requirements for publication in Data in Brief as stated in https://www.elsevier.com/authors/journal-authors/policies-and-ethics.

## CRediT Author Statement

**Mehmet Evren Soylu:** Conceptualization, Investigation, Formal analysis, Methodology, Validation, Visualization, Writing – original draft; Writing – review & editing; **Rafael L. Bras:** Supervision, conceptualization, Writing – review & editing.

## Declaration of Competing Interest

The authors declare that they have no known competing financial interests or personal relationships that could have appeared to influence the work reported in this paper.

## Data Availability

Global Distribution of Shallow Groundwater (Original data) (Hydroshare).
